# Two years of psychogeriatric consultations in a nursing home: reasons for referral compared to psychiatrists' assessment

**DOI:** 10.1186/1472-6963-6-73

**Published:** 2006-06-13

**Authors:** Camilla MT Callegari, Marco Menchetti, Giovanni Croci, Scilla Beraldo, Chiara Costantini, Federico Baranzini

**Affiliations:** 1 Dipartimento di Medicina Clinica-Psichiatria, Università degli Studi dell'Insubria, via Rossi 9, 21100 Varese, Italia; 2 Istituto di Psichiatria, Università degli Studi di Bologna, via Pepoli 5, 40123 Bologna, Italia

## Abstract

**Background:**

In spite of the high prevalence of psychiatric disorders among elderly residents in nursing homes, only a small number of patients in need of specialist care are referred to a psychiatric consultant. The aim of this research was to evaluate the consultation activity and the appropriateness of referral to psychiatric assessment.

**Methods:**

Data were collected and analysed on consultation carried out over a two-year period in a RSA (Residenza Socio-Assistenziale) in Northern-Italy. Data were catalogued with reference to: patients, consultation, diagnosis and recommended medications. Statistical correlation analysis by means of Spearman test and signification test was carried out.

**Results:**

Residents referred to psychiatric consultation at least once were 112 (14.5% of all residents). Reason for referral were: depression (17.2%), delusions and hallucinations (14%), agitation (34.8%), aggressive behaviour (23.5%) and disturbances of sleep (6.8%). Most frequent diagnoses were organic, including symptomatic, mental disorders (33.9%), mood disorders (22.3%) and schizophrenia, schizotypal and delusional syndromes (18.8%). No psychiatric diagnosis was found only in 1.8% of cases, thus confirming high sensibility of referring physicians.

A statistically significant correlation was found when comparing referrals for depression or delusions and allucinations or sleep disturbances and diagnostic confirmation of such symptoms by specialistic assessment (respectively 49.8%, 52.7% and 19.6%).

Correlation between psychotic symptoms and the consequent prescription of antipsychotic drugs had a significant if somewhat modest value (24%) while correlation between depression symptoms and prescription of antidepressant drugs was more noticeable (66.5%).

**Conclusion:**

Main reason for referral to psychiatric consultation resulted to be the presence of agitation, a non-specific symptom often difficult to attribute. Data concerning depression confirm tendency to underestimating this diagnosis in the elderly. Furthermore, symptomatic reasons for referral did not always correspond to subsequent diagnostic definitions by psychiatric consultants, therefore demonstrating modest predictive power.

## Background

A number of epidemiological studies have demonstrated a high prevalence of psychiatric disorders among elderly residents in nursing homes: almost half, in fact, present symptoms of depression which are clinically significant [1-3] and an extremely high percentage of these, varying between 14 – 26%, come into the category of major depression disorders [2,4]. Psychotic symptoms, such as delusions and hallucinations, behavioural disturbances and agitation are presented with the same frequency [2,5]. Furthermore, most of these disturbances are associated with the presence of cognitive deterioration or dementia [6] and chronic medical pathologies which increase the level of disability, thus making the psychiatric diagnosis and the correct treatment of the patient extremely difficult. In fact, if hyperactive delirium is often recognized among patients referred for agitation and behavioural disturbances, hypoactive delirium is, on the contrary, undetected [7] because its symptoms are less disturbing and do not represent a reason for psychiatric referral.

Psychiatric disorders represent an important part of the workload for the personnel working in these structures, however only a small number of patients who are in need of specialist care are referred to a psychiatric consultant [8-10]. It has, in fact, been noted that symptoms of depression are often not correctly recognised [11] as they may merely be interpreted as inevitable signs of old age or cognitive deterioration, or as they are less disturbing within the community environment are considered irrelevant and are therefore underestimated.

Considering the high prevalence of psychiatric disorders among elderly residents in nursing homes and the difficulty of treating them, psychiatric referral in these structures would appear to be crucial in order to improve both the assistance and the quality of the last remaining years of life.

The aim of this research was to evaluate the consultation which takes place in a residential nursing home for the elderly in Lombardy and to verify the pertinence of the request for psychiatric evaluation by comparing the reason for the request and the diagnosis made by the specialist. The characteristics of the consultation carried out were evaluated both in terms of motivation and urgency for the required intervention, and also in relation to diagnosis and recommended medications.

## Methods

The study was carried out in the 'Residenza Sanitaria Assistenziale (RSA) Fondazione Molina' in Varese, a state funded home providing assistance and rehabilitation for the elderly who for the most part are no longer self-sufficient. It is composed of four large units and has 430 beds. There is also an integrated Day Care Centre.

The personnel consists of doctors specialized in internal medicine and geriatrics, professional nurses, physiotherapists, psychomotor therapists, welfare assistants, social workers, occupational therapists and social service care officers. National Health specialists are called in by the ward doctor to conduct any necessary consultations.

Since March 2000 psychiatric consultations have been in the hands of the Psychiatric Unit of the Department of Clinical Medicine of the Università degli Studi dell'Insubria.

A request for psychogeriatric referral is made by the internal medical staff, by filling out a special form giving: patient's name, location, date of the request, type of specialist examination, level of urgency and reason for the request. The examinations are carried out by the psychiatric specialist accompanied by post-graduate students in psychiatry.

Data were collected and analysed for examinations carried out over a two year period, between 27^th ^March 2000 and 27^th ^March 2002.

The collection of data was done manually both from the request forms and the patients' notes kept in the departments, for the in-patients and in the archives for those discharged or deceased. Data were catalogued according to:

• **patients: **age, sex and level of disability classified in the patient's notes as Totally Non Self-Sufficient (NAT), Partially Self-Sufficient (NAP) and Self-Sufficient (AUTO);

• **referral: **reason for psychiatric referral given by the ward doctor; type (urgent, ordinary, not specified)

• **symptomatology**: symptoms collected by consulting psychiatrist.

• **formal diagnosis: **made according to the ICD-10 classification by the consultant specialist in psychiatry;

• **recommended therapy: **psychopharmacological, psycho-rehabilitative, psychological support.

The reasons given, in the terminology expressed on the forms received, have been broken down into five groups:

1. depression

2. delusions and hallucinations

3. agitation

4. aggressive behaviour

5. insomnia

Any reasons different from the above, being only sporadic (e.g. anxiety, control, therapy re-evaluation, etc.) were put into a category named 'other' (they were not, however, included in the data study). In some cases the reason for the request was only one, in others more than one.

During the 2 year period of the study there were no psychiatric requests for dementia. This was probabilly caused by the fact that in the nursing home Fondazione Molina patients with diagnosis of dementia were mainly referred to the neurologist. Furthermore most of the residents with this diagnosis reside in a specific Alzheimer unit.

For the statistical analysis, a correlation was established between the reason for the request and the symptomatology elicited from the psychiatrists' assessment. For this purpose the latter was, then, grouped into five classes of psychiatric symptoms:

1. agitation

2. psychotic symptoms

3. symptoms of depression

4. cognitive deterioration

5. sleep disorders

For the statistical analysis of correlations we used sintomatology ascertained by the consultant instead of formal diagnosis classified according to the ICD-10, because symptoms gave a wider spectrum of elderly psychiatric clinical conditions and behaviours.

The correlation study was carried out using the Spearman test (ρ), binominal correlation test for nominal/categorical variables and the signification test.

The software SPSS was used for the elaboration of data.

## Results and discussion

### Characteristics of the sample

The total number of residents in the Fondazione Molina during the period under consideration was 775 – 564 female (72.8%) and 211 male (27.2%). Residents referred to psychiatric consultation at least once were 112 (14.5% of the reference population). This percentage is the same as that which emerges from publication data [9,12,13], and appears to confirm the tendency to underestimate the real prevalence of psychiatric pathology among the elderly in residential care homes.

Most of the residents (82.1%) for whom psychiatric referrals were requested were totally non self-sufficient (NAT). The sample composition demonstrates the distribution according to age and gender of the total population in the home, as shown in Table 1.

### Type of referral requested

During the 2 year period, 224 consultations were carried out, 176 were for women (78%) and the remaining 48 for men (22%). The average age of the patients requiring consultation resulted as being 79.69 years (SD ± 9.231) for women and 77.13 years (SD ± 10.441) for men.

Table 2 show the percentage distribution and frequency whitin gender (M/F). It demonstrates that most of the residents for whom a psychiatric consultation had been requested belonged to the geriatric age (conventionally fixed at ≥ 65). 12.5% was outside this age range (Fondazione Molina under special circumstances also admits persons < 65 y.o.). When evaluating the age distribution of the consultations, it may be noted that the 65–84 age group is particularly effected and that the percentage for both sexes (52.2% F, 52.1% M) is the same. In the age group ≤64 years the consultations mainly applied to men (20.8% M vs 10.3% F), vice-versa in the age group ≥ 85 years (37.5% F vs 27.1% M).

Thus for the latter age group we can resume that 37 female patients (12% of the female residents) required 66 consultations (1,78 pro capite) while 9 male patients (12% of the male residents) required 13 consultations (1,44 pro capite).

Taking into account the number of consultations carried out during the two year period per single patient, three categories may be distinguished:

1. patients receiving only one consultation: 69 (61.6%)

2. patients receiving 2 consultations: 20 (17.9%)

3. patients receiving 3 or more consultations (up to a maximum of 11): 23 (20.5%).

The type of consultation was divided as follows:

• Urgent: 51 (22.8%)

• Ordinary: 161 (71.9%)

• Not specified: 12 (5.3%).

**Figure 1:**
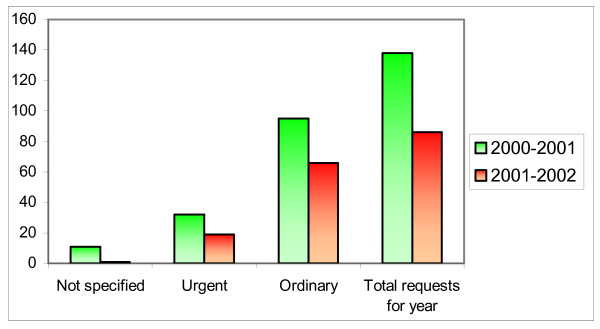
Type of consultation: distribution of the type of request during the two years of evaluation.

Figure 1, on comparing the absolute number of consultations carried out during the first year (2000–2001) with that of the second (2001–2002), demonstrates a considerable drop in the number of requests for specialist psychiatric examinations. One explanation for this data has been hypothesized that after the psychiatric consultations had been placed in the hands of university personnel from the Psychiatry Unit of the Università dell'Insubria, the ward doctors had chosen to include a larger number of patients for the evaluation of the new specialist. Thus allowed the psychiatrist to get to know the residents with previous clinical histories of psychiatric disturbances. The reduction in the number of requests for consultations during the second year can also be interpreted as a better selection of cases, a consequence of an improvement in specialist activity. To support this hypothesis there is also a conspicuous reduction in the percentage of request catalogued as 'not specified' between the first and second year of activity (from 8% to 1.2%) which would further confirm a better level of collaboration.

Reasons for requests for consultation from the residential home are distributed as follows: depression (17.2%), delusions and hallucinations (14%), agitation (34.8%), aggressive behaviour (23.5%) and sleep disturbances (6.8%). Can be noted a greater frequency of agitation and aggressive behaviours that represent both operational and relational problems within the residential units.

**Figure 2:**
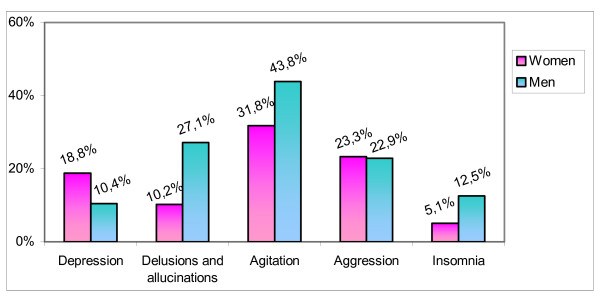
Reason for request and gender: distribution of reasons for referral requests by gender.

Figure 2, which shows the distribution of the reasons for the request by gender, reveals that the percentage for aggressive behaviours is analogous in both sexes, depression is higher in females and other motivations are more prevalent among males. Nevertheless, no statistically significant differences were found in the distribution of the type of request by gender or age, with the exception of a higher frequency of requests concerning psychotic symptoms (delusions and hallucinations) in men (p < 0.0004).

**Figure 3:**
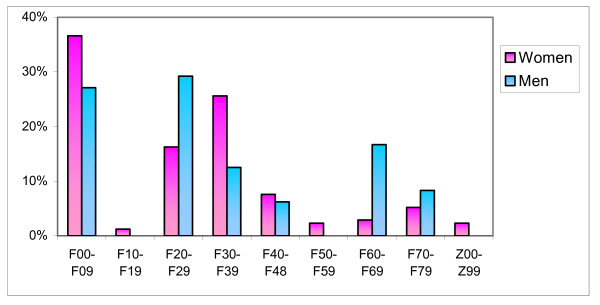
Diagnosis and gender: distribution by gender of the diagnoses made by the psychiatric specialist.

**Figure 4:**
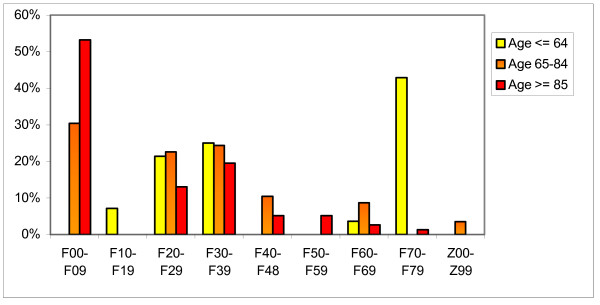
Diagnosis and age: distribution by age of the diagnoses made by the psychiatric specialist.

Figures 3 and 4 represent the distribution by gender or age of the diagnoses formed according to the ICD-10, the percentage values on the total sample, grouped into the following categories are:

• Organic, including symptomatic, mental disorders (F00-09): 33.9%,

• Mental and behavioural disorders due to psychoactive substance use (F10-19): 0.9%,

• Schizophrenia, schizotypal and delusionals disorders (F20-29): 18.8%,

• Mood [affective] disorders (F30-39): 22.3%,

• Neurotic, stress-related and somatoform disorders (F40-49): 7.1%,

• Behavioural syndromes associated with physiological disturbances and physical factors (F50-59): 1.8%

• Disorders of adult personality and behavioural (F60-69): 5.8%,

• Mental retardation (F70-79): 5.8%.

Only in 1.8% was no psychiatric diagnosis made, thus confirming a good capability of discerning when a psychiatric examination was in fact necessary.

As shown in Figure 3, in women there was a more frequent diagnosis for organic, including symptomatic, mental disorders (36.6% vs. 27.1% in men) and mood disorders (25% vs. 12.5%). The frequency of diagnosis in the schizophrenia and delusional syndrome category resulted much higher in males (29.2% vs. 16.3% in females), as also the diagnosis of personality and behavioural disorders (18.7% against 2.9%). In the older age groups there is a larger number of psychic disorders of an organic nature and syndromes and behavioural syndromes associated with physiological disturbances and physical factors (Figure 4). For the age ≤ 64 these pathological conditions are absent while there is a clear prevalence for the diagnosis of mental retardation, probably due to a premature institutionalization of these patients.

### Correlations between reason for the request and diagnoses

**Figure 5:**
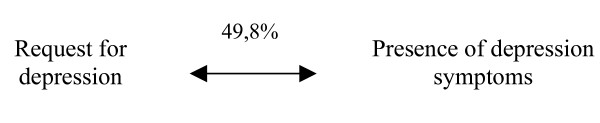
Request for depression: correlation concerning requests for depression.

A statistically significant correlation was found (ρ = 0.498; ρ < 0.001) when comparing referrals for depression and diagnostic confirmation of such symptoms by specialistic assessment (Figure 5). If calculated excluding cases in which the request was made for sleep disturbances, agitation or aggressive behaviour this value increases, although only slightly (ρ = 0.525). That would suggest that these factors could be confusing for the non specialized personnel. The referrals for suspected depression not judged as such by the consultant were only 4. More numerous (42) were the referrals in which the psychiatrist ascertained symptoms of depression but the request had been made for other reasons. This would indicate, in accordance with data in literature [11,14,15], that depression symptomatology is often underestimated.

**Figure 6:**
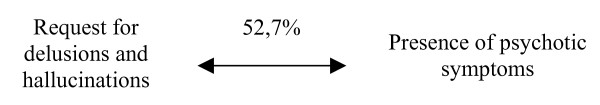
Request for psychotic symptoms: correlation concerning requests for delusions and hallucinations.

Also the correlation between the request for delusions and hallucinations and the actual symptoms of the schizophrenic series ascertained by the psychiatrist during the consultation, demonstrate a statistically significant value (ρ = 0.527; ρ < 0.001) (Figure 6). This data may be influenced by a frequent presence in the elderly of psychotic symptoms of major depression (melancholic delusion) or hallucinations and delirium during the course of a physical illness (hallucinations and delirium). It is probable that the psychotic symptoms present in these forms are not recognized as such by the ward doctors.

The slight, even though statistically significant (ρ = 0.411; ρ < 0.001) correlation of data concerning agitation may likewise be attributed to the scarce specificity of this condition.

**Figure 7:**
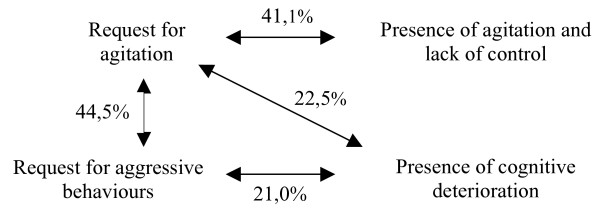
Request for agitation: correlations concerning agitation, aggressive behaviours and cognitive deterioration.

An extremely noticeable correlation was found between the presence of cognitive deterioration and the symptom of agitation recognised by the psychiatrist (ρ = 0.444; ρ < 0.001), while more moderate values are evident when the cognitive deterioration is related to agitation (ρ = 0.225; p < 0.002) or aggressive behaviour (ρ = 0.210; p < 0.003) codified as a reason for request (Figure 7).

A strong correlation has also emerged between the request concerning agitation and that for aggressive behaviour (ρ = 0.445; p < 0.001) confirming the fact that a state of agitation may be a prelude to aggressive attitudes.

This data are in line with those in literature which indicate agitation as one of the most common behavioural complications of dementia which worsens with the advancement of cognitive impairment. [16].

**Figure 8:**
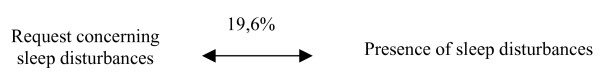
Request for sleep disturbances: correlation concerning sleep disturbances.

Another statistically significant correlation is that between the requests for sleep disturbances and the ascertained presence of theese simptoms (ρ = 0.196; p < 0.005) (Figure 8). This is probably caused by those patients for whom the request was made for reasons other than the subsequent objectiveness of sleep function disorders.

### Psychopharmacological therapy

During the two year period in question psychopharmacological therapy was indicated in 92.4% of the consultations carried out. These were: 79.2% antipsychotics (NL typical and atypical), in 52.7% anxiolytics and sleep inducers (BDZ), in 45.4% antidepressants (TCA, SSRI, SNRI), in 26.1% anti-Parkinson (anticholinergics) and in 10.6% mood stabilizers (carbamazepine, gabapentin). In 7.6% no therapy was administered.

In most cases a combination of drugs was prescribed belonging either to the same or different categories. Furthermore, the non medical indications, although not strictly quantifiable, represent an invaluable tool in the therapeutic approach to the patient. Patients were frequently referred to the psychomotor therapists and/or physiotherapists and talks with relatives, support psychologists, social assistants were customary. Discussion groups were periodically organized with members of staff from the different professions taking part.

**Figure 9:**
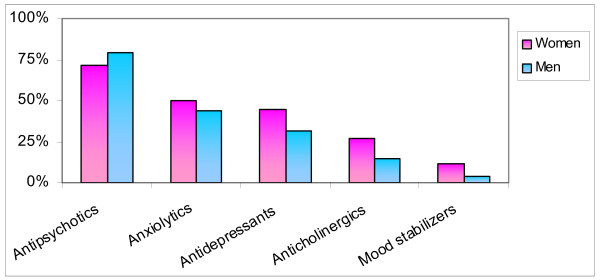
Therapy and gender: distribution of psychopharmacological therapies by gender.

**Figure 10:**
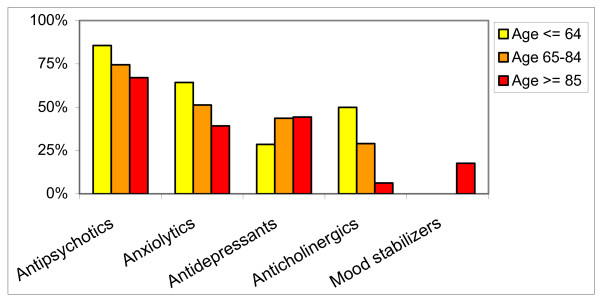
Therapy and age: distribution of psychopharmacological therapies by age.

As far as the psychopharmacological prescriptions are concerned no statistically significant differences were found between men and women for any pharmaceutical category (Figure 9). When taking into consideration the distribution by age (Figure 10), however, it can be noted that with the increase in age there is a reduction in the use of antipsychotics, anticholinergics, and benzodiazepines. For these last two categories of drugs there does in fact exist a statistically significant difference in the distribution by age (p < 0.001 and p < 0.04 respectively), which on the other hand is not evident in the case of the antipsychotics. On the contrary, however, the use of antidepressants increases, although not in a statistically significant way, in the more advanced age groups and the use of mood stabilisers is present only in the ≥ 85 age group (p < 0.02).

**Figure 11:**
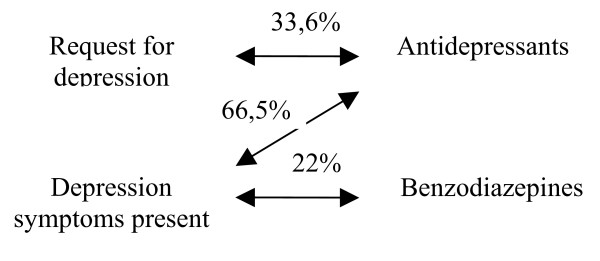
Depression and therapy: correlations concerning depression and psychopharmacological therapies.

In the analysis carried out regarding the pathology of depression (Figure 11) a statistically significant correlation was ascertained:

• comparing depression symptoms ascertained by the specialist and the prescription of antidepressant drugs (ρ = 0.665;p.< 0.001) and/or benzodiazepines (ρ = 0.222;p < 0.002);

• comparing depression as reason for the referral on the consultation request form and the prescription by the psychiatrist of antidepressant drugs (ρ = 0.336; p < 0.001).

A statistically significant correlation also exists, but in the negative, between the presence of depression symptoms and the use of antipsychotic drugs (ρ = -0.327; p < 0.001).

The correlation between psychotic symptoms and subsequent use of antipsychotic drugs has a significant value (ρ = 0.240; p < 0.001) as also that between reasons for delusions and hallucinations on the consultation request and the use of this same pharmacological category (ρ = 0.149; p < 0.03). Furthermore, the consultant, due to the advanced age of many patients and the frequent co-morbidity with somatic pathology, takes great care when selecting cases to be treated with neuroleptics. Moreover, some psychotic symptoms, if they are an expression of a general medical condition, tend to regress as the basic illness itself improves.

**Figure 12:**
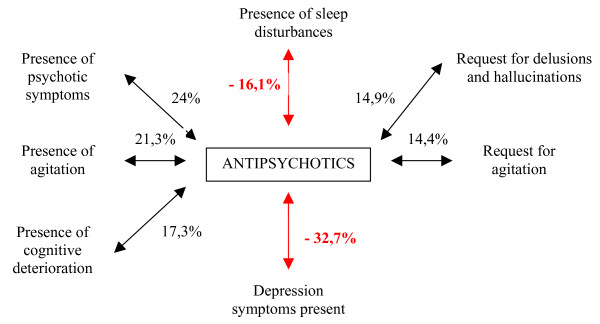
Antipsychotics: correlations concerning the use of antipsychotics.

A statistically significant correlation was found when comparing prescriptions of antipsychotics and consultation requests by the ward doctors concerning agitation (ρ = 0.144; p.<0.04) (Figure 12). Instead the correlation between antpsychotics prescriptions and reasons for request concerning aggressive behaviours does not appear to be significant. If, on the other hand, the states of agitation confirmed by specialistic assessment are taken into consideration, it is significant both in relation to the use of antipsychotics (ρ = 0.213; p < 0.003) and, although with lower values, of mood stabilizers (ρ = 0.139; p < 0.05). In particular, regarding the use of antipsychotics, it has emerged that this pharmacological category is used in cases of cognitive deterioration (ρ = 0.173; p < 0.02) but not for sleep disturbances (negative correlation, ρ = -0.161; p < 0.03) (Figure 12).

**Figure 13:**
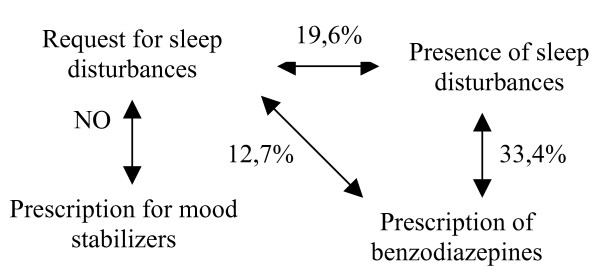
Sleep disturbances and therapy: correlations concerning sleep disturbances and psychopharmacological therapies.

On examining the pharmacological intervention adopted in the case of sleep disturbances it can be noted that benzodiazepines are the most commonly recommended (ρ = 0.334; p < 0.001) while no statistically significant correlation was ascertained between reasons regarding sleep disturbances and psychiatrist's prescription of mood stabilizer drugs (Figure 13).

**Figure 14:**
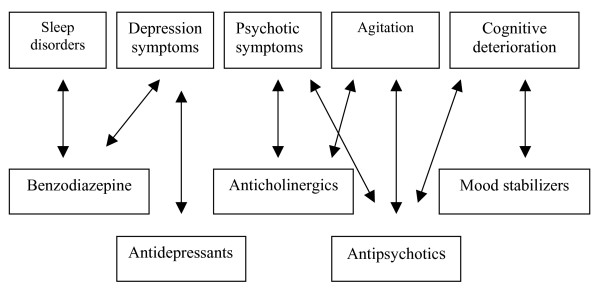
Symptomatology and therapy: resume of correlations concerning symptomatology ascertained during psychiatrists'assessment and psychopharmacological therapies.

Finally a positive correlation has been noted between the presence of cognitive deterioration and the use of mood stabilizers (ρ = 0.222; p < 0.002), and antipsychotics (Figure 14), and a negative one with the use of benzodiazepines (ρ = -0.150; p.<0.03).

From the therapeutic indications supplied by the psychiatrist during consultations it was also noted that:

• a positive correlation exists between the use of antidepressants and anxiolytics (ρ = 0.231; p < 0.002) and a negative correlation between the use of antidepressants and antipsychotics (ρ = -0246; p < 0.001).

• a statistically significant correlation exists between the use of anti-Parkinson and antipsychotics (ρ = 0.335; p < 0.001), and between anti-Parkinson and anxiolytics (ρ = 0.239); p.0.001), while the correlation between anticholinergics and mood stabilizers is statistically significant but in the negative (ρ = -0159; p < 0.02).

## Conclusion

From the results obtained it has emerged that the main reason for psychiatric consultation requests for the elderly in nursing homes is the presence of agitation, associated or not with aggressive behaviours. The fact that this symptom is absolutely aspecific makes it even more difficult to interpret. Agitation can, in fact, masque somatic problems, which are even life-threatening (cardiac disturbances, neoplasia), likewise it may be the only symptom of a depression syndrome such as melancholy. It is also important to consider that behavioural correlations of agitation in the elderly are often expressions of the difficulty to adapt to life in an institution. A change of environment, in these cases, can represent a traumatic element in the life of an elderly person, worsened by feelings of uselessness and frustration that come to light over a period of time spent in residential care. Because clinical signs are not always immediately diagnosed and the necessity for immediate therapeutic intervention, agitation can often provoke reactions of alarm and destabilization. This problem is accentuated even further by the difficulties which arise when managing the patient. Furthermore this disturbing behaviour generates intolerance among the nursing staff, the other residents and relatives who have difficulty in understanding the cause of such a condition. Agitation, consequently, is seen by the nursing staff, more of a hindrance to normal activities rather than the symptom of a possible illness and therefore a request for psychiatric intervention for the patient also includes a request concerning back-up for the medical team that has to cope with the case.

Data concerning depression pathology underline how too often it can be underestimated in the elderly patient. In fact, the patients with symptoms of depression do not provide a disturbing element within the residential community, consequently the attention of the nursing staff is not drawn towards them, and cases often remain undiagnosed. Not infrequently the medical staff do not recognize or underestimate these symptoms because they are considered an inevitable expression of the ageing process or cognitive deterioration. The elderly suffering from depression thereby disappear into the routine of health care often continuing with antidepressants prescribed by their doctors before entering the nursing home with no further therapeutic revaluation being requested. These cases frequently only emerge on extreme occasions: the patient refuses to eat, threatens suicide, is agitated or delirious. It is also true that a somatic illness or accompanying cerebral damage make diagnosis more difficult, especially when observing that in many cases elderly patients suffering from depression masque mood swings with somatic complaints or clinically evident manifestations.

In conclusion this study underlines how the real necessity for nursing home residents to receive psychiatric consultation must be evaluated in a pertinent way by the doctors during ward rounds.

Nevertheless, symptoms indicated as the reason for consultation do not always correspond to those found by the specialist and therefore give only a modest predictive indication of the final diagnosis formulated. According to our findings, the complexity of problems encountered in the environment of the nursing home can not be totally captured by diagnostic categories, but is better conveyed by the description of symptoms both reported from staff and found during specialistic assessment. Diagnoses 'per se' can allow to segregate patients into defined categories but do not seem to be an adequate basis for understand real needs of patients and for planning a clinically helpful therapeutic program.

In this context psychogeriatric consultation aims to improve the integration of somatic and psychological aspects, in order to provide a global and personalized approach for each individual patient.

## Competing interests

The author(s) declare that they have no competing interests.

## Authors' contributions

CC conceived of the study, drafted the manuscript and revised it.

MM participated in the design and coordination of the study.

GC made substantial contributions to acquisition and interpretation of data.

SB partecipated in drafting and revising the manuscript.

CC collected the data, performed the statistical analysis and was involved in drafting the manuscript.

FB made substantial contributions to conception and design of the study and interpretation of data.

All authors read and approved the final manuscript.

## Pre-publication history

The pre-publication history for this paper can be accessed here:



## Figures and Tables

**Table 1:** sample distribution and nursing home residents distribution according to gender and age.

**Patients**		**Age ≤ 64**	**Age 65–84**	**Age ≥ 85**	**Total**
**Sample**	**Women**	4 (4.8%)	43 (51.2%)	37 (44.0%)	84 (100%) (75%)
	**Men**	5 (17.9%)	14 (49.9%)	9 (32.2%)	28 (100%) (25%)
	**Total**	9 (8%)	57 (50.9%)	46 (41.1%)	112 (100%) (100%)
**Nursing home residents**	**Women**	31(5.5%)	225 (39.9%)	308 (54.6%)	564 (100%) (72.8%)
	**Men**	19 (9.0%)	117 (55.5%)	75 (35.5%)	211 (100%) (27.2%)
	**Total**	50 (6.5%)	342 (44.1%)	383 (49.4%)	775 (100%) (100%)

**Table 2:** distribution of consultations according to gender and age.

**N of consultations**	**Age ≤ 64**	**Age 65–84**	**Age ≥ 85**	**Total**
**Females**	18 (10.3%)	92 (52.2%)	66 (37.5%)	176 (100%) (78%)
**Males**	10 (20.8%)	25 (52.1%)	13 (27.1%)	48 (100%) (22%)
**Total**	28 (12.5%)	117 (52.2%)	79 (35.3%)	224 (100%) (100%)
